# Results of PCR Analysis of Mpox Clinical Samples, Sweden, 2022

**DOI:** 10.3201/eid2906.230253

**Published:** 2023-06

**Authors:** Jon Edman-Wallér, Ola Jonsson, Gustav Backlund, Shaman Muradrasoli, Klara Sondén

**Affiliations:** Sahlgrenska University Hospital, Gothenburg, Sweden (J. Edman-Wallér);; University of Gothenburg Institute of Biomedicine, Gothenburg (J. Edman-Wallér);; Linköping University Hospital, Linköping, Sweden (O. Jonsson);; Falu Hospital, Falun, Sweden (S. Muradrasoli);; Public Health Agency of Sweden, Solna, Sweden (S. Muradrasoli, K. Sondén);; Karolinska Institute Department of Medicine, Solna (K. Sondén)

**Keywords:** mpox, monkeypox virus, MPXV, viruses, PCR, cycle threshold, Ct, rectal samples, skin lesion samples, Sweden

## Abstract

We compared cycle thresholds from mpox skin lesions with other specimen sites and over time from onset of clinical signs among 104 patients in Sweden. Cycle thresholds differed by anatomic site. We identified 2 early mpox cases from anorectal swab specimens after skin samples were negative, indicating necessity of sampling multiple sites.

A global outbreak of mpox was detected in May 2022 and declared a Public Health Emergency of International Concern on July 23 (*1*). We investigated how monkeypox virus (MPXV) cycle threshold (Ct) values, a proxy for viral loads, differed on the basis of specimen type and duration of illness among patients in Sweden. Ethics approval was deemed unnecessary because all analyses were based on anonymized laboratory data available from the Public Health Agency of Sweden. 

## The Study

As of February 19, 2023, Sweden had 260 confirmed mpox cases; peak incidence was in August 2022. During the May 24–September 2 study period, most cases were diagnosed at the Public Health Agency of Sweden. As in other countries, most cases were found among men who have sex with men. In most cases, the disease has been self-limiting, and skin lesions are the most common clinical sign (*2*). 

Samples were collected from all parts of the country from patients with suspected mpox disease based on clinical observation; for this study, we included 289 samples from 104 patients with >1 positive test. We used in-house real-time PCR targeting the B21 gene of MPXV for diagnostic testing of all specimen types. Before setup, we tested analytic specificity in vitro against cowpox and vaccinia viruses and in silico against other orthopoxviruses. 

For the analyses, we included specimen type, Ct value, and days since onset of clinical signs as variables for each sample. When multiple samples from the same patient, day, and sample site existed, we analyzed the sample with the lowest Ct. We tested Ct values from skin lesions compared with paired samples from other specimen sites from the same patient and date for statistical significance using Wilcoxon signed-rank test. We used R Core Team version 4.2.2 software (The R Project for Statistical Computing, https://cran.r-project.org) for statistical analyses and to generate graphs. 

**Figure 1:**
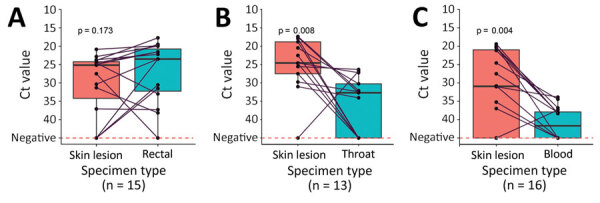
Mpox Ct levels of paired tests from different specimen types collected on the same day from the same patient and analyzed at the Public Health Agency of Sweden, May 24–Sep 2, 2022. Black dots indicate Ct levels of individual samples and gray lines link results from different specimen sites on the same day from the same patient. Ct, cycle threshold.

The World Health Organization recommends MPXV PCR testing primarily from skin lesions (*3*), which have higher sensitivity than other specimen types (*4*); skin lesions were the most common specimen type in our study. (Table). Previous studies comparing Ct from paired skin lesion and oropharyngeal samples have usually shown lower mean values in skin samples but lower values from the oropharynx in some individual cases (*5*). In our study, all but 1 skin lesion sample had lower Ct values than oropharyngeal samples taken on the same day (Figure 1). Semen was positive in 4/6 samples. Within the cohort of patients with same-day rectal and skin samples of which >1 was positive (n = 15), we found no significant difference in Ct values between samples from the 2 sites. This finding agreed with previous research (*3*), but only a minority of patients (n = 22) were tested, and those might have had prominent clinical signs from the rectal area (e.g., perianal lesions or proctitis). 

**Table:** Mpox PCR tests, specimen types, and cycle threshold levels for 104 patient samples analyzed at the Public Health Agency of Sweden, May 24–September 2, 2022*

Specimen type	Samples				Ct values of positive samples				Patients	
	Total no.	No. positive	Positive, %		Median	25th percentile	75th percentile		Total no.	≥1 positive samples
Skin lesion	178	148	83.1		23.6	20.6	28.6		96	92
Rectum	22	21	95.5		23.0	21.0	31.2		22	21
Throat	16	10	62.5		30.9	27.5	32.3		15	10
Blood†	17	8	47.1		37.6	36.0	38.0		8	7
Semen	6	4	66.7		32.9	31.1	35.2		5	3
Nasopharynx	13	4	30.8		34.9	32.8	36.6		5	3
Saliva	10	8	80.0		35.1	32.0	37.0		3	3
Sputum	8	5	62.5		31.2	30.2	35.0		4	2
Urine	7	2	28.6		30.3	28.6	32.0		5	2
Other	3	1	50.0		20.6	20.6	20.6		2	1
Unknown	9	4	50.0		22.1	21.2	23.1		4	2

*Ct, cycle threshold. †Blood includes whole blood, plasma, and serum samples.

**Figure 2:**
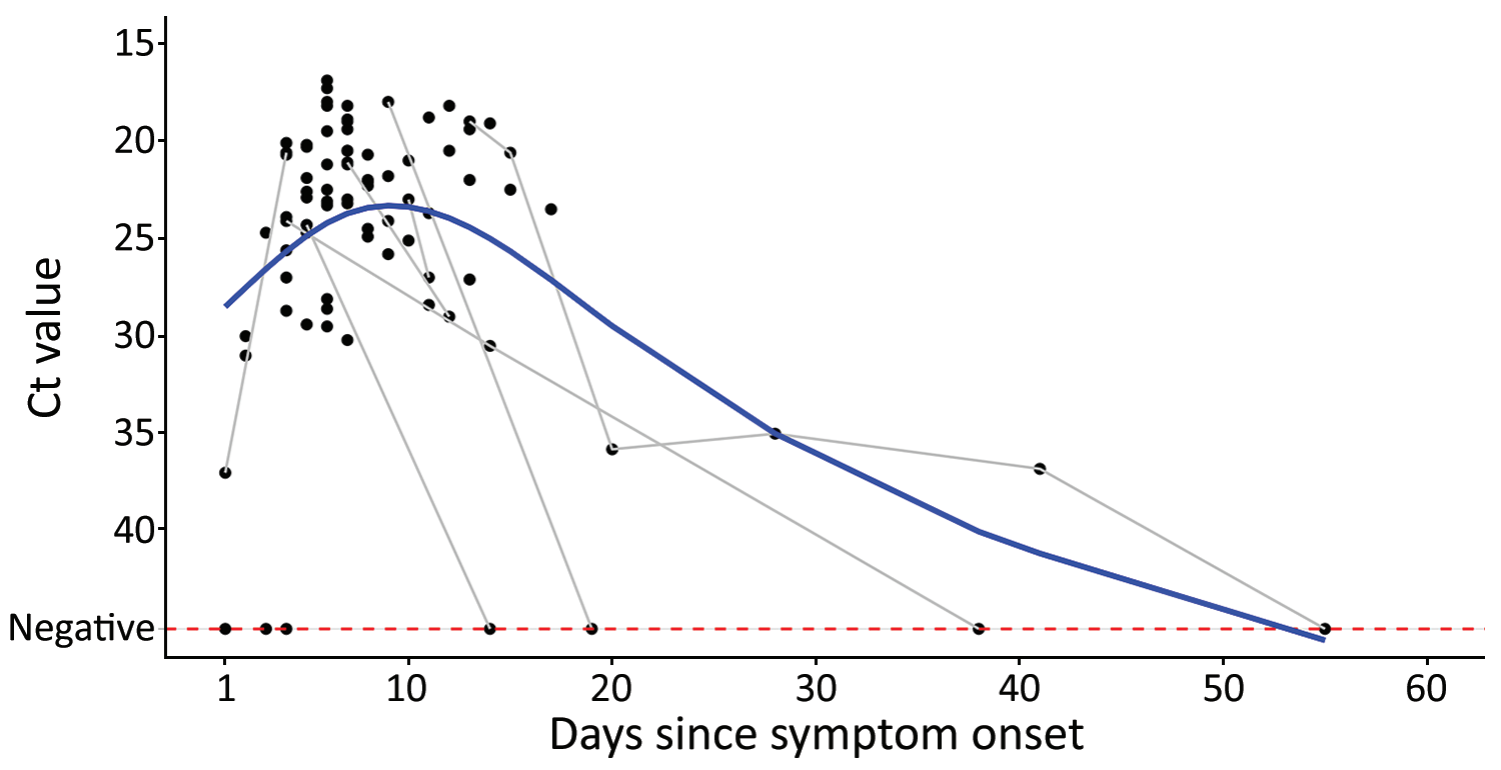
Mpox PCR Ct values in skin lesion samples and days since onset of signs (day 1) analyzed at the Public Health Agency of Sweden, May 24–Sep 2, 2022 (n = 83). Gray lines connect samples from the same patient. Blue line (smoothing spline) is for visualization.

Ct values were lowest from skin samples taken ≈1 week after onset of signs (Figure 2). Post hoc analysis showed significantly higher Ct values from samples taken on days 1–5 after onset of signs (n = 36, median Ct = 28.2, interquartile range [IQR] 23.8–37.3) than on days 6–10 (n = 58, median = 23,2, IQR 20.7–28.3; p = 0.004), possibly because vesicular or pustular lesions that develop after a few days might have been easier to sample or have a higher actual viral load. After the initial decrease, Ct values from skin lesions increased over time (Figure 2). At 16 days after onset of signs, >50% of skin lesion samples were PCR negative. This period is in line with the clinical course documented elsewhere, in which infection usually resolves 2–4 weeks after the onset of rash (Z.M. Afshar et al., unpub data,https://doi.org/10.22541/au.165446104.43472483/v1), although cases of prolonged disease and PCR positivity have been described elsewhere (*6*). Previous research has also shown generally decreased viral loads 14 days after the first positive test (*7*). 

Among 83 patients with skin lesion samples for whom data on onset of signs were available, 3 were negative based on first skin lesion samples. However, 2 of those patients were found positive from rectal samples taken the same days as the corresponding skin sample (days 3 and 4 after onset of signs) and 1 was positive in a serum sample (day of onset of signs). Those results suggest that rectal and serum testing may be more useful early in the disease course, when skin lesions are at an early stage. In addition, in 2 patients for whom dates of onset of signs were unavailable and who were found negative from first skin lesion samples, we found 1 was positive from a rectal sample and the other from a throat swab sample from the same days. 

## Conclusions

Our data on MPXV PCR tests during the mpox 2022 outbreak in Sweden indicate that viral levels peak in skin lesions 6–10 days after onset of signs and samples may test negative in early disease. Mpox detection rates might increase with complementary anorectal sampling in early disease or repeated skin lesion testing when patients with suspected lesions test negative soon after onset of signs. A strength of the study was the national coverage, but because final Ct levels varied with sampling methods, extraction methods, and PCR targets, and detailed clinical data were missing in this study, our results need to be confirmed in other settings. 
